# Reasons for participating in randomised controlled trials: conditional altruism and considerations for self

**DOI:** 10.1186/1745-6215-11-31

**Published:** 2010-03-22

**Authors:** Sharon K McCann, Marion K Campbell, Vikki A Entwistle

**Affiliations:** 1 Health Services Research Unit, University of Aberdeen, Aberdeen, UK; 2 Social Dimensions of Health Institute, University of Dundee, Dundee, UK

## Abstract

**Background:**

Randomised controlled trials of healthcare interventions depend on the participation of volunteers who might not derive any personal health benefit from their participation. The idea that altruistic-type motives are important for trial participation is understandably widespread, but recent studies suggest considerations of personal benefit can influence participation decisions in various ways.

**Methods:**

Non-participant observation of recruitment consultations (n = 25) and in-depth interviews with people invited to participate in the UK REFLUX trial (n = 13).

**Results:**

Willingness to help others and to contribute towards furthering medical knowledge featured strongly among the reasons people gave for being interested in participating in the trial. But decisions to attend recruitment appointments and take part were not based solely on consideration of others. Rather, they were presented as conditional on individuals additionally perceiving some benefit (and no significant disadvantage) for themselves. Potential for personal benefit or disadvantage could be seen in both the interventions being evaluated and trial processes.

**Conclusions:**

The term 'conditional altruism' concisely describes the willingness to help others that may initially incline people to participate in a trial, but that is unlikely to lead to trial participation in practice unless people also recognise that participation will benefit them personally. Recognition of conditional altruism has implications for planning trial recruitment communications to promote informed and voluntary trial participation.

**Trial registration:**

ISRCTN15517081

## Background

Randomised controlled trials of healthcare interventions depend on the participation of volunteers who might not be expected to derive any personal health benefit from the interventions being evaluated (beyond that which they might reasonably expect to derive from healthcare outside the trial). It is therefore widely assumed that altruistic-type motives are important for trial participation. But because trial participants might not derive any personal health benefit from their participation, and because trial participation can bring with it various burdens, it is widely recognised that potential volunteers should be carefully informed about the trial and enabled to consider the personal implications of participation before deciding whether or not to volunteer.

Many trials recruit fewer participants than anticipated [1-3], although the feasibility of recruiting sufficient participants to yield useful results is usually carefully estimated. A study of recruitment in a cohort of 114 UK multicentre trials funded by the UK Medical Research Council and the UK Health Technology Assessment Programme between 1994 and 2002 found that fewer than a third (31%) achieved their original recruitment target and half (53%) were awarded an extension. The proportion achieving targets did not appear to improve over time [4].

Concern to improve trial recruitment processes to facilitate the participation of sufficient numbers of appropriately informed volunteers has helped to stimulate an interest in studies of patients' perspectives on trial recruitment processes and trial participation. A number of rigorous qualitative investigations have generated important insights into factors that can influence trial participation, the understandings (and misunderstandings) that people have about (aspects of) trials that they (are asked to) participate in, and the retrospective reflections and evaluations of participation experiences [3,5-28].

Alongside this primary research, we also conducted a meta-ethnographic synthesis of qualitative studies of potential volunteers' accounts of issues influencing their decisions about trial participation. The meta-ethnographic synthesis is being written up for separate publication, but we note briefly here that 12 studies published prior to 2006 met our inclusion criteria [3,6,7,9,12,18,19,21-23,26,27]. These were considered chronologically and the concepts identified as salient by the original authors were extracted and compared. We grouped the concepts into four key concept groups relating to: a) personal circumstances at the time of trial entry; b) views about the interventions being compared in the trial; c) views about trial processes and procedures; and d) the 'weighing up' of possible benefits to self and to others of participating in the trial.

We contribute to and extend this literature with our report of a study that aimed primarily to explore patients' perspectives on recruitment to and participation in a multi-centre trial (the REFLUX trial) that compared medical and surgical interventions for patients with gastro-oesophageal reflux disease (GORD), and that included parallel patient preference arms within its design. We paid particular attention to people's accounts of their decisions about whether or not to participate.

### The REFLUX trial

The REFLUX trial was set up to assess the effectiveness and cost effectiveness of minimal access surgery compared with continued optimised long term medical management amongst people with GORD [29]. GORD is a chronic condition that causes some of the most frequently seen symptoms in both primary and secondary care; between 20% and 30% of 'Western' patients experience heartburn and/or reflux intermittently [30-32]. Reflux is the backflow of acid from the stomach into the oesophagus and occurs when the valve at the lower end of the oesophagus does not function properly. The usual symptom is heartburn, and for some people, reflux can become frequent and severe enough to require daily medication for symptom control.

The treatment of GORD includes both medical and surgical options. Medical management involves stepped therapy resulting in the daily use of proton pump inhibitors (PPIs) [33]. Once started on PPIs, the majority of patients with significant GORD remain on long-term treatment [34,35]. Whilst PPIs are generally assumed to be safe, there are concerns regarding their long-term use [36].

The role of surgery in treatment of GORD is contentious and ill defined [37]. Currently patients only have access to anti-reflux surgery if their long-term medication is failing to control their symptoms. Although surgery seems highly effective for controlling GORD in the short-term, it is unclear whether the benefits of surgery remain long term.

The REFLUX trial focused on people with GORD whose symptoms would otherwise be managed with long term therapy with PPIs. Patients were eligible for the REFLUX trial if they already required maintenance medical therapy, had documented evidence of GORD, symptoms for more than 12 months, were suitable for either medication or surgery; and the recruiting clinician was uncertain as to which management policy was better for the patient.

Although the REFLUX trial was primarily a RCT, it also included parallel non-randomised patient preference groups in recognition that the marked differences in the treatments were likely to make the choice between them strongly preference sensitive. Patients who refused randomisation because of strong preferences (for medication or surgery) were allowed to access their treatment of choice and be followed up within the study [38,39]. Within the partially-randomised patient preference trial design, potential participants who did not express strong treatment preferences were randomised in the conventional way. However, for logistical reasons and to maintain a balance between the sizes of the preference and randomised groups, the number of participants that could be recruited to the preference arms was restricted to 20 participants per intervention in each recruitment centre. When this quota was reached, patients could only participate in the randomised arm of the trial. The trial, which was conducted in 21 centres across the UK, recruited a total of 357 randomised participants (178 surgical, 179 medical) and 453 preference participants (261 surgical, 192 medical).

Participants in the medication arm of the trial continued on their PPIs with no further tests or follow up appointments required. In contrast, participants in the surgical arm of the trial had to undergo additional investigative procedures (pH manometry test, and an endoscopy procedure if they had not had one previously), and attend an outpatient appointment with the surgeon prior to the anti-reflux surgery. Following surgery, participants required between two and six weeks off work for recovery. All participants were asked to complete baseline and follow up questionnaires.

The trial was conducted from the Health Services Research Unit, University of Aberdeen, and funded by the NHS Research and Development (now NIHR) Health Technology Assessment Programme. Full MREC and R&D approval was received.

## Methods

This qualitative study was embedded in the REFLUX trial. It aimed to examine in-depth people's experiences of trial recruitment and participation. A combination of methods was used: non-participant observations of recruitment appointments, followed by in-depth interviews with the people who were deemed eligible and invited to participate in the REFLUX trial.

Between April 2003 and May 2004, SM approached a total of 26 people in two trial recruitment centres (Centres A and B to preserve anonymity) to join this qualitative study. Only one person declined to participate. SM observed the recruitment appointments of the 25 people who consented (13 in Centre A and 12 in Centre B). She made field notes of key observation points during and after the appointments, but did not audio- or video-record the consultations. The observations were used to give the research team insight into what was happening in practice in recruitment appointments, to help identify issues to explore with potential trial participants in interviews, and to provide contextual information to support interpretation of the interview data.

Eleven of the people whose consultations were observed (5 in Centre A and 6 in Centre B) were judged by the recruiting clinicians to be ineligible for the REFLUX trial. They were subsequently excluded from this qualitative study.

Of the 14 people deemed eligible for REFLUX participation, one subsequently declined to be interviewed. For the remaining 13 people SM arranged face-to-face interviews (regardless of whether or not they agreed to participate in the REFLUX trial).

Interviews were conducted by SM, either in participants' homes, at the recruitment hospital or the study research office. All but two took place approximately one week after recruitment appointments (two participants asked to be interviewed immediately after their recruitment appointments). The interviews aimed to explore people's experiences of the recruitment process and to identify the factors that they thought had influenced their decisions to attend the recruitment appointment and then to accept or decline trial entry. The interviews were conversational in style, but SM used a topic guide to help ensure that each person was invited to describe the story of their GORD and its treatment to date, to describe and reflect on each stage of the recruitment process (letter of invitation or advert; scheduling of recruitment appointment; recruitment visit; communication with members of the trial team after the recruitment appointment); to express their opinions about trial recruitment communications; and to discuss what they had thought about and what had been influential in their decisions to participate or not in the trial.

At the time of these interviews, participants had consented or declined to take part in the REFLUX trial, but those who had agreed to randomisation did not know the treatment to which they had been allocated.

The research ethics committee required that interviewees be sent a copy of their transcript to read and offered an opportunity to suggest any amendments they wished to make. None of the interviews suggested any amendments to their transcripts. Data analysis started from the first observation and was an ongoing process that informed data collection in the later stages of the study (mainly by suggesting issues to probe in more detail in later interviews) [40]. Data were analysed thematically with the assistance of a computerised qualitative data analysis package (QSR Nvivo 2.0) [41].

The first stage involved familiarisation with the data. Initial (open) codes were generated, discussed among the team and grouped according to the stages of recruitment processes that they referred to and/or into thematic categories relating to the types of experiences, considerations or reasons that seemed salient from participants' perspectives. The second stage of analysis involved developing and refining thematic categories, and examining the relationships within and between them, bearing in mind the chronological sequences within individual accounts. SM led the analysis and undertook the systematic coding of transcripts. MC and VE contributed to the generation and refinement of open codes and thematic categories, and to the consideration of relationships that resulted in the findings presented here. In the illustrative data extracts below, names of centres, doctors and participants have been replaced by numbers and letters to preserve anonymity. As part of the consent process, participants were informed that the results of the study would be published in academic journals.

## Results

Five women and eight men were interviewed for this study. Five of them had been taking prescribed acid-suppression medication for GORD for at least five years. Some still had poor symptom control, and some had concerns about taking long-term medication. Most were prescribed their acid-suppression medication by a general practitioner, and several had not consulted a specialist about their GORD (prior to the REFLUX trial recruitment appointment).

Nine of the thirteen people interviewed had agreed to be randomised; two had agreed to join the preference medical arm of the trial (both in centre B) and two had declined to take part in the trial (both in centre A). (The preference arm was not available in centre A at the time of this study as its quota for preference participants was reached before SM started observing clinics). Table 1 summarises the characteristics of the 13 study participants.

**Table 1:** Summary characteristics of qualitative study participants

Participant number	Gender and approximate age of participants	Duration of prescribed medication at time of trial entry	Centre	Trial participation decision
**1**	Female early 40 s	8 years	A	Randomised

**2**	Female early 50 s	5 years	A	Declined

**3**	Female mid 40 s	10-13 years	A	Randomised

**4**	Male mid 60 s	6 years	A	Randomised

**5**	Male early 20 s	2-3 years	B	Preference medical

**6**	Male early 30 s	4-5 years	B	Randomised

**7**	Male early 50 s	18 months	B	Preference medical

**8**	Female early 60 s	18 months -2 years	A	Declined

9*	Female early 20 s	12 -18 months	B	Randomised

**10**	Male late 30 s	1 year	A	Randomised

**11**	Female mid 50 s	8 years	A	Randomised

**12**	Male early 50 s	1 year	B	Randomised

**13**	Male mid 40 s	1 year	B	Randomised

**14**	Male late 40 s	18 months - 2 years	A	Randomised

*Participant 9 initially consented to joining the qualitative study and to participate in the REFLUX trial. However, she did not respond to any subsequent communications and so contributed no data to the study.

When discussing their decisions about whether or not to attend a REFLUX trial recruitment appointment and whether or not to participate in the trial, people mentioned a number of considerations and portrayed these as having been more or less influential. Our main findings were (i) that willingness to help others and to contribute towards furthering medical knowledge featured strongly among the reasons people gave for being interested in participating in the trial, but (ii) decisions to take part were also presented as conditional on individuals additionally perceiving some benefit (and/or no significant disadvantage) for themselves. In this section, we focus first on the initial willingness to help others and then describe the ways in which people considered they might either benefit or not benefit personally from attendance at a REFLUX trial recruitment appointment and from participation in this trial.

**Figure 1:**
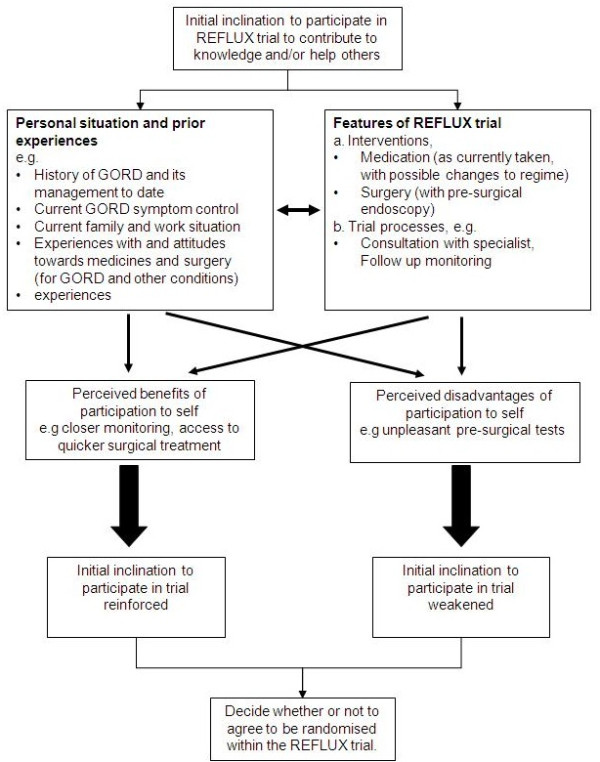
**Schematic overview of considerations reported to influence participation in the REFLUX trial**.

A schematic overview of the ways in which these main categories of consideration were described as having influenced participation in the REFLUX trial is presented in Figure 1.

### a) Initial inclination to participate to help others

In interviews, people explained that they had been motivated to attend the trial recruitment appointment and possibly take in the trial at least in part because they had an inclination to help others or help generally:

"The reason I wanted to try it is because, they're obviously trying to find the recommended solution to heartburn or which is the best way for doing it.....if I can help in any way to sort of find where the best way forward it, then I was quite keen to just help out and do that." (Centre B/Dr C/Pt 6/randomised surgical)

"It sounds corny, but I just want to be of some assistance....I could have taken the easy option, which is always the easy option, just to say 'No, I'm not interested'. But nobody benefits from that." (Centre A/Dr B/Pt 14/randomised surgical).

However, this initial willingness was not necessarily their only motivator to attend a trial recruitment appointment, and it was not presented as a sufficient reason for taking part in the trial. In the next two sections, we consider perceptions of potential for personal benefit that tended to encourage participation and perceptions of potential for personal disadvantage that tended to discourage it.

### b) Potential to benefit personally

People identified a number of ways in which they might benefit personally from attending the initial recruitment appointment and (possibly) from taking part in the trial. These were strongly related to their understandings of their current situation and of the health care options available to them outside the trial.

#### • Trial recruitment appointments as an opportunity for learning and review

Several people mentioned that the trial recruitment appointment had presented an opportunity to have their condition and treatment reviewed by a specialist, and/or for them to learn more in the context of a personal consultation than they could by general reading about GORD. For example:

"I just thought it would be a good chance to discuss things, em, find out more for myself, and then also help other people in the future with the same sort of thing....it was as much selfish reasons as wanting to help other people! Cos I wanted to find out more information, em I find you get better information, you know, face-to-face conversation than looking at random studies on the internet..." (Centre B/Dr C/pt 5/preference medical)

Our observations revealed that information provision by clinicians and question-asking by patients were both quite variable across recruitment appointments. Some clinicians gave the impression of being very rushed, while others worked through discussions and examinations at a steadier pace and seemed to be more thorough. Not all patients asked questions when offered the opportunity to do so.

#### • Trial participation potentially offering access (and faster access) to surgery

Most of the people who had agreed to be randomised within the REFLUX trial indicated at some point in their interviews for this study that they were hoping to be allocated to surgical treatment because this represented an opportunity to possibly improve their symptoms (if these were not alleviated by medication alone) and/or to stop taking long term medications. For example:

"You know, I've already had tablets for 8 years, so, I just hope, well I hope, that that comes into it.....I am hoping that I'll be randomised to get an operation." (Centre A/Dr A/Pt 1/randomised medical)

"I'll be perfectly honest, I would probably choose to go on the surgery, em, because I've been on tablets for a while, and, intermittently been off the tablets and the symptoms keep coming back, it would be interesting possibly to try alternative method to see if that's going to be a success for me personally." (Centre B/Dr C/Pt 6/randomised medical)

None of the people who expressed a preference for surgery in the interviews for this study had been observed to disclose a preference for surgery in their recruitment appointments, and observation notes record that some had explicitly said that they had no preference between the treatments or were undecided which would be best for them.

Several of the people who were inclined to favour surgery had also thought they might access surgery more quickly in the context of the REFLUX trial. They saw this as a reason in favour of participation. For example:

"One of my reasons for, I would like to take part in it, I would think, I might maybe get some quicker treatment? Because I had gallbladder trouble nine years ago and I waited a long time for any treatment, and I was in a lot of pain, so, maybe...I thought that I would get quicker treatment." (Centre A/Dr A/Pt 1/randomised medical)

We did not observe any evidence of clinicians suggesting in recruitment appointments that people allocated to the surgical arm of the trial would be treated more quickly than people receiving surgery outwith the trial.

#### • Trial participation offering careful monitoring

A couple of interviewees said they thought their condition would be more closely 'monitored' if they participated in the REFLUX trial, and that this had increased their inclination to participate. For example:

"Well again, it's going to be monitored over the years isn't it?....in this study they are gonna keep their eye on you...I mean you go along to change your medication but somehow there may well be more thought put behind it, you know, the monitoring. Whereas your doctor doesn't have time, you know? " (Centre A/Dr A/Pt 11/randomised medical)

The expectation of closer monitoring in the context of the trial might have derived from a prior general understanding of what trials entail, or perhaps from the participant information leaflet, which explained that a number of questionnaires would be sent to trial participants. We did not observe any discussion of the potential benefits (or disadvantages) of trial participation in terms of follow-up assessments for individual patients in the trial recruitment appointments.

### c) Personal concerns or lack of potential for personal benefit from trial participation

The two study participants who declined to take part in the REFLUX trial both described how initially they had wanted to help others and had considered participating for this reason. However, their accounts suggest their initial willingness to participate had lessened as they learned more about the trial during the recruitment appointment and developed concerns about negative implications for themselves - especially if they were allocated to the surgery arm. Both of these people had good symptom control with medication. One was apprehensive about undergoing an unpleasant pre-surgical test (pH test) and the other thought the risks of undergoing surgery were unwarranted given her current condition:

" No the keyhole surgery, no that doesn't bother me, it's just the thought of that test." (Centre A/Dr A/Pt 8/declined trial entry)

"I would have liked to have helped with the research and what have you, but em, I just didn't feel I was ready for it [surgery] at this moment in time...I know it's keyhole surgery, but things can go wrong with keyhole surgery...and I feel that unless I'm in an awful lot of pain I'm quite willing to take the medication to keep it under control, rather than having the operation. In the future if I'm still having problems, and I probably think it's the right thing to do I would probably opt for the surgery, but only much further down the line if that was really the only option left...but I'm reluctant right now..What if it doesn't work out, would things end up worse off than they are just now?." (Centre A/Dr A/Pt 2/declined trial entry)

For these people, an initial inclination to participate in the trial did not lead on to trial participation because of concerns that participation would involve exposing themselves to unnecessary potential harms. The two people in centre B who declined to be randomised but agreed to take part in the preference medical arm of the REFLUX study expressed similar concerns about the pre-surgical tests and surgery.

## Discussion

The main findings of this study were that people reported having an inclination to help others or contribute to a collective general good that predisposed them towards trial participation, but that considerations of the implications of trial participation for them personally also featured in their accounts as influential reasons for or against participation. For the people who agreed to be randomised, trial participation seemed to be something of a "win:win" situation - one in which they could both help others and benefit (or at least not be harmed) personally. Both *trial processes *(such as attending recruitment appointments that included specialist assessments, and undergoing additional symptom monitoring as part of trial follow up) and *trial interventions *(such as pre-surgical tests and surgery) were considered by potential participants as sources of benefit or harm. Some participants, however, had misconceptions about these.

Our study had several strengths. We conducted observations as well as interviews, and the interviews about trial recruitment experiences were conducted within a week of trial recruitment appointments. Our study is one of the first to examine potential participants' perspectives on trial recruitment in the context of trial that had patient preference arms. However, we only recruited participants from two trial recruitment sites, and only one of these was still offering participation in preference arms by the time we were able to conduct interviews. Our sample size, and the number of participants who declined randomisation but accepted follow up in a preference arm, were therefore limited.

This study is not the first to show that people cite a desire or willingness to help others among the factors motivating their participation in clinical trials, [1,3,12,13,16,27] nor, indeed to show that perceptions of potential for individual benefit also feature in their considerations [7,9,10,12-15,17,28]. However, our findings help illuminate the relationship between consideration for others and considerations for self in the context of trial participation.

When consideration of others motivates action, it is sometimes referred to as 'altruism'. 'Altruism' is a contested concept, not least because of the multiple challenges of differentiating considerations of others from considerations of self. Opinions vary as to whether the term should only be used when consideration of others is the sole or overriding motivator, and/or whether it should only be used when personal interests are somehow harmed at the same time as others' or collective interests are promoted.

These and other theoretical and practical issues have led to the development of a number of variants to the basic concept of altruism [42,43]. In the context of clinical trials, the term 'weak altruism' [44] has been proposed to describe a situation where patients consent to participate only because they perceive 'no positive net difference' between treatments and so do not expect to lose out. Canvin and Jacoby [13] drawing on their study of people being invited to take part in an epilepsy trial, suggest that this term can be usefully extended to describe the situation where people are "happy to help others, but only where they could also help themselves."

We suggest that the term 'conditional altruism' might more accurately describe what was observed in our study. It summarises the finding that although people may initially have a tendency to participate in a trial based on a willingness to help others or contribute to a general good, this is unlikely to lead to trial participation in practice unless people can also recognise that trial participation can benefit (or at least not harm) themselves in ways that they regard as salient.

Our study findings also add to the body of evidence that suggests people's decisions about whether or not to take part in trials are sometimes influenced by misconceptions about how, why and to what extent they might benefit personally from participation [5,7,9,18,23,26] - even when they have been given information materials that conform to current trial regulations and have spoken with clinically qualified trial staff. They suggest there is no room for complacency in trial recruitment communication. They also tend to confirm that people for whom a current standard treatment is not working well are likely to regard trial participation as a positive opportunity to access a new intervention that could be better [21], and that people who agree to be randomised may harbour preferences for particular treatments that trialists are unaware of [5]

### Implications for trialists

The notion of 'conditional altruism' draws attention to the idea that while altruistic tendencies can encourage trial participation, such participation is still conditional on perceptions of personal benefit and an absence of overriding concerns. This raises some important questions about how trialists should communicate with (potential) participants - especially given the evidence that (potential) participants may hold or develop misconceptions about the ways in which they might benefit from or be disadvantaged by trial participation.

Assuming that the goals of recruitment communications are to invite and encourage people to participate in trials for which they are eligible, but also to ensure that their decisions about participation are based on an understanding of what is at stake and are congruent with their own values and good reason, our findings suggest that trialists need to strive to enable people to understand and consider the various ways in which both others and themselves might benefit, as well as the various ways in which their own interests might be poorly served by trial participation. Trial recruitment communication might usefully enable people to consider whether they are in a "win: win" situation in which both they and others might benefit from their participation.

The promotion of consideration for others as a reason for participating in research does raise ethical issues but will not necessarily be inappropriate [45]. Others' interests may be seen as bound up with self interest in various ways - not least when satisfaction or social credit are derived from promoting the interests of needy or deserving others. The promotion of consideration of potential for personal benefit from trial participation is also ethically sensitive, particularly in the light of awareness that people sometimes have unrealistic ideas about what they might gain and how from trial participation.

Clearly, trialists should not over-state the case for any potential for personal benefit that might be derived from trial participation. Our study suggests that more positive steps need to be taken to ensure both collective and personal issues are considered, and to avoid and address any potential misconceptions about the implications of trial participation. This includes misconceptions that people 'bring into' trials or 'develop' from what they are told - some of the misconceptions observed in this study could not be simply and directly attributed to trial recruitment materials or consultations.

A number of strategies might be adopted. The types of misconception that have been identified in several studies might now be anticipated, and pre-trial discussions or research with members of the groups of people who will be eligible for trial participation could also help identify any new or specific misunderstandings that might arise in the context of particular trials, and the ways in which potential participants' hopes or fears might tend to be unduly heightened. Once identified, these issues can be addressed explicitly in information materials [46].

Trial recruitment decision aids might also be useful as a means of enabling and encouraging potential participants to engage with information and consider the key implications of their trial participation options. A decision aid that was developed to improve the consent process for women invited to participate in a breast cancer prevention trial was recently reported to have been helpful to the women as they considered trial participation, and it resulted in a greater understanding compared to reading a participant information sheet [47]. However, trialists who develop recruitment decision aids will need to work through a range of issues. Decisions about how to structure the trial participation options (especially when there are preference arms to be considered), how to present trial interventions and procedures, and which potential collective and personal issues to mention and associate with which option features will all need to be carefully considered [48].

The potential importance of two-way interpersonal communication for the support of informed trial participation decisions that take both social and personal considerations into account should not be underestimated. The variability of recruitment consultations observed and participant understandings expressed in our relatively small study support the need to identify and cultivate more effective approaches to communication among trial recruitment staff as well as to attend to more standardised information resources. Decision aids that encourage people to indicate preferences and discuss these with their clinicians or trial recruitment personnel might also help expose uncertainties or misunderstandings that could then be addressed before decisions about participation were finalised. But with or without these, careful discussions of what individuals hope to contribute to others and gain or avoid for themselves could be crucial to help avoid situations in which people's decisions to participate are based at least in part on misunderstandings about their potential to benefit [49].

## Conclusions

Willingness to make a contribution to the collective good and to help others is commonly thought to be - and relied upon as - a key motivating factor for participation in clinical trials. However, the relationship between a willingness to help others and other factors that might bear on trial participation is complex. The term 'conditional altruism' concisely captures the insights from this study that although willingness to help others might generally incline people towards participation, participation is still conditional on expectations of benefiting personally to some extent. A more informed understanding of motivations to participate in a trial, and of the kinds of beliefs that underpin these, could usefully underpin the knowledge and practice of trial recruiters as they attempt to grapple with the complexities of trial recruitment and make judgements about how best to support people making decisions about trial participation.

## Competing interests

The authors declare that they have no competing interests.

## Authors' contributions

SM, MC and VE all made substantial contributions to the conception and design of the study. SM conducted all the fieldwork and all authors contributed to the analysis and interpretation of data. All authors participated in the drafting of the manuscript. All authors read and approved the manuscript.
